# L-Type Calcium Channels Contribute to Ethanol-Induced Aberrant Tangential Migration of Primordial Cortical GABAergic Interneurons in the Embryonic Medial Prefrontal Cortex

**DOI:** 10.1523/ENEURO.0359-21.2021

**Published:** 2022-01-28

**Authors:** Stephanie M. Lee, Pamela W. L. Yeh, Hermes H. Yeh

**Affiliations:** Department of Molecular and Systems Biology, Geisel School of Medicine at Dartmouth, Hanover, NH 03755

**Keywords:** alcohol, calcium, calcium channels, FASD, GABA, interneuron

## Abstract

Exposure of the fetus to alcohol (ethanol) via maternal consumption during pregnancy can result in fetal alcohol spectrum disorders (FASD), hallmarked by long-term physical, behavioral, and intellectual abnormalities. In our preclinical mouse model of FASD, prenatal ethanol exposure disrupts tangential migration of corticopetal GABAergic interneurons (GINs) in the embryonic medial prefrontal cortex (mPFC). We postulated that ethanol perturbed the normal pattern of tangential migration via enhancing GABA_A_ receptor-mediated membrane depolarization that prevails during embryonic development in GABAergic cortical interneurons. However, beyond this, our understanding of the underlying mechanisms is incomplete. Here, we tested the hypothesis that the ethanol-enhanced depolarization triggers downstream an increase in high-voltage-activated nifedipine-sensitive L-type calcium channel (LTCC) activity and provide evidence implicating calcium dynamics in the signaling scheme underlying the migration of embryonic GINs and its aberrance. Tangentially migrating Nkx2.1^+^ GINs expressed immunoreactivity to Cav1.2, the canonical neuronal isoform of the L-type calcium channel. Prenatal ethanol exposure did not alter its protein expression profile in the embryonic mPFC. However, exposing ethanol concomitantly with the LTCC blocker nifedipine prevented the ethanol-induced aberrant migration both *in vitro* and *in vivo*. In addition, whole-cell patch clamp recording of LTCCs in GINs migrating in embryonic mPFC slices revealed that acutely applied ethanol potentiated LTCC activity in migrating GINs. Based on evidence reported in the present study, we conclude that calcium is an important intracellular intermediary downstream of GABA_A_ receptor-mediated depolarization in the mechanistic scheme of an ethanol-induced aberrant tangential migration of embryonic GABAergic cortical interneurons.

## Significance Statement

The etiology of fetal alcohol spectrum disorders (FASD) takes place *in utero* when the fetus is exposed to alcohol. While the outcome of FASD has been well characterized, the mechanism underlying its embryonic etiology is incompletely understood. Here, we investigated the role of L-type voltage-gated calcium channels (LTCCs) in the ethanol-induced aberrant tangential migration of cortical GABAergic interneurons (GINs). The findings from our study highlight LTCCs as important regulators underlying the aberrant tangential migration resulting from prenatal ethanol exposure and suggest that they bear therapeutic potential in managing and treating FASD. The results also propose an interplay between chloride and calcium in the migrating embryonic interneurons, and exposure to ethanol may enhance this interaction, contributing to the etiology of FASD.

## Introduction

Fetal alcohol spectrum disorders (FASD), hallmarked by lifelong physical, behavioral, and intellectual abnormalities, is the leading cause of preventable neurodevelopmental disorders (American Academy of Pediatrics, 2000; Carr et al., 2010; Mattson et al., 2011). Alcohol (ethanol) readily crosses the placenta and can be detected in the fetus as well as in the amniotic fluid (Idänpään-Heikkilä et al., 1972; Cuzon et al., 2008). The National Survey on Drug Use and Health reported that, from 2015 to 2018, drinking prevalence for pregnant women in the past 12 months was 64.7%, current drinking was 9.8%, and current binge drinking was 4.5% (England et al., 2020). Of those respondents in their first trimester of pregnancy, 19.6% reported current alcohol use and 10.5% reported binge drinking. Many women are not aware that they are pregnant until after the fourth or sixth week of pregnancy, increasing the risk of ethanol consumption early in gestation (Floyd et al., 1999). The prevalence of FASD is estimated at 33.5 per 1000 live births in the United States, 22.7 globally, and as high as 113.22 in some populations (Roozen et al., 2016). These statistics underscore that FASD is a significant public health concern. Understanding its embryonic etiology is thus critical for managing and treating FASD.

We have employed a mouse model of FASD in which pregnant mice consumed ethanol either chronically throughout gestation or in a binge-type pattern to investigate the effects of prenatal ethanol exposure on the development of the cerebral cortex (Cuzon et al., 2008; Skorput et al., 2015; Delatour et al., 2019, 2020). Ethanol exposure *in utero* throughout gestation altered tangential migration of corticopetal GABAergic interneurons (GINs), regulated by an ambient level of GABA in the embryonic telencephalon (Cuzon et al., 2006, 2008). Binge-type ethanol exposure also disrupted tangential migration, which was associated with a persistent interneuronopathy that manifested as an excitatory/inhibitory (E/I) imbalance, hyperactivity, and deficits in executive function in young adult mice (Skorput et al., 2015). In a more recent study (Skorput et al., 2019), acute exposure to ethanol enhanced the membrane depolarization mediated through GABA_A_ receptors expressed in GINs migrating in the embryonic telencephalon. This ethanol-induced enhancement was normalized by bumetanide, a blocker of the NKCC1 co-transporter. Co-treatment of pregnant dams with bumetanide and ethanol also mitigated aberrant tangential migration in the short term, as well as interneuronopathy and behavioral deficits in the long-term. However, it remained unclear how the potentiation of GABA-mediated depolarization following ethanol exposure disrupted tangential migration. Here, we hypothesized that this ethanol-potentiated depolarization triggers downstream signaling molecules that enhance migration, notably an increase in the activity of voltage-gated calcium channels which, in turn, leads to an abnormally augmented level of tangential migration.

Indeed, calcium signaling is a key mechanism underlying neuronal migration, including tangential migration of cortical GINs (Komuro and Rakic, 1996, 1998; Soria and Valdeolmillos, 2002; Moya and Valdeolmillos, 2004; Inada et al., 2011). Migrating neurons undergo a wide range of cellular processes, including neurite extension, nucleokinesis, and trailing tail retraction through cytoskeletal dynamics, all of which are regulated by calcium-dependent signaling (Gomez and Spitzer, 1999; Lautermilch and Spitzer, 2000; Gomez et al., 2001; Wen et al., 2004; Kerstein et al., 2017). Calcium influx through L-type voltage-gated calcium channels (LTCCs) has been implicated in various processes of neuronal and non-neuronal migration, including neurite extension and trailing tail retraction (Yang and Huang, 2005; Darcy and Isaacson, 2009; Bortone and Polleux, 2009; Danesi et al., 2018; Kamijo et al., 2018). Neurotransmitters such as glutamate and GABA have been reported to invoke calcium influx through LTCCs (Bortone and Polleux, 2009; Horigane et al., 2019). Furthermore, LTCCs have been linked to neuropsychiatric disorders, including autism spectrum disorders, timothy syndrome and schizophrenia (Cross-Disorder Group of the Psychiatric Genomics Consortium, 2013; Kabir et al., 2017; Danesi et al., 2018).

In the present study, we tested the hypothesis that an ethanol-induced potentiation of LTCCs contributes to the aberrant tangential migration of GINs. We report here that co-treatment with ethanol and nifedipine, an LTCC blocker, prevented the ethanol-induced aberrant tangential migration both *in vitro* and *in vivo*. In addition, we report that ethanol potentiates LTCC activity in migrating GINs in the embryonic cortex. We conclude that LTCCs play an important role in manifesting the aberrant migration of embryonic GINs induced by prenatal ethanol exposure and propose that they may be potential therapeutic targets for mitigating the teratogenic effects of ethanol on the pathoetiology of FASD.

## Materials and Methods

### Animals

All procedures were performed in accordance with the National Institutes of Health *Guide for the Care and Use of Laboratory Animals* and approved by Dartmouth College Institutional Animal Care and Use Committee. The Nkx2.1-Cre transgenic mouse line (originally obtained from Stewart Anderson; Xu et al., 2008) was crossed with the Ai14 Cre-reporter mouse line (The Jackson Laboratory) to yield Nkx2.1Cre/Ai14 mice harboring tdTomato-fluorescent medial ganglionic eminence (MGE)-derived GINs (referred to as Nkx2.1^+^ GINs; Skorput et al., 2015). For time-pregnant mating, pairs of male and female mice were housed overnight, with the following day designated as embryonic day (E)0.5. E13.5–E16.5 embryos were included in this study as available. Based on results of Y-chromosome-specific genotyping (Stratman et al., 2003), no difference in the ratio of male to female offspring was noted between treatment groups. E13.5–E16.5 were operationally defined to be within the age range roughly equivalent to mid-first trimester in humans (Clancy et al., 2001; Parnell et al., 2014).

### Ethanol exposure paradigm

This study used the binge-type ethanol exposure paradigm reported in previous studies (Skorput et al., 2015, 2019; Delatour et al., 2019, 2020). Timed-pregnant mice were exposed to ethanol from E13.5 to E16.5, when tangential migration of MGE-derived cortical interneuron is at its peak in mice (Parnavelas, 2000; Anderson et al., 2001; Marín and Rubenstein, 2001; Jiménez et al., 2002; Batista-Brito and Fishell, 2009; Gelman et al., 2009; Hladnik et al., 2014). Pregnant dams were individually housed and fed a liquid diet (Research Diets) containing alcohol (5% w/w) or isocaloric control diet containing maltose. Mice were maintained under normal 12/12 h light/dark cycle and water was available *ad libitum*. The liquid food was replenished daily between 9 and 11 A.M., when the amount consumed and the weight of the dams were measured. Following termination of the liquid diet on E16.5, the mice were returned to standard chow. Mean dam blood alcohol level (BAL), measured at 11:30 P.M. on E15.5, was 0.08%, consistent with previous binge ethanol measurements (Skorput et al., 2015, 2019). Blood was collected via tail-vein and assessed using an Analox Instruments GM7 series analyzer. Following the binge-type ethanol consumption paradigm, pregnant dams carried their offspring to full term with no apparent effect on litter size. For nifedipine treatment, nifedipine stock was dissolved in DMSO and added to the control or 5% ethanol-containing liquid food to achieve 0.15 mg of nifedipine per kilogram of body weight of the mice being fed. The final dilution of DMSO in the liquid food was 1:120,000.

### Organotypic embryonic slice culture

Organotypic culture of embryonic slices was performed as described previously (Cuzon et al., 2006; Skorput et al., 2019). At E14.5, time-pregnant dams were asphyxiated using CO_2_ and embryos were removed by cesarean section. The tdTomato-expressing embryos were identified using fluorescence UV (Biological Laboratory Equipment Maintenance and Service). The brains were quickly harvested under a dissecting microscope and immersed in ice-cold slicing media (1:1 F12:DMEM) oxygenated by continuous bubbling with 95% O_2_/5% CO_2_. The dissected brains were embedded in 3.5% low melting point agarose in 1:1 F12:DMEM, and 250-μm-thick coronal slices were collected using a vibrating microtome (Electron Microscopy Sciences) in ice-cold slicing media with 95% O_2_/5% CO_2_.

The organotypic slices were placed on a fine nylon mesh supported on a U-shaped platinum wire in a 35-mm round Petri dish. To achieve air-liquid interface, 0.8-ml sterile filtered culture media (1:1 F12/DMEM, 1% penicillin/streptomycin, 1.2% 6 mg/ml glucose in DMEM, 10% fetal bovine serum, and 1% L-glutamine) was added. After the slice cultures were incubated in a humidified incubator (37°C, 5% CO_2_) for 1 h, sister cultures were randomly replenished with culture medium containing: (1) 6.5 mm EtOH, (2) 20 μm nifedipine, (3) 6.5 mm EtOH + 20 μm nifedipine, and (4) 0.02% DMSO. We used this low concentration of ethanol for the organotypic slice culture experiment as it is equivalent to the blood alcohol concentration (30 mg/dl) observed in a mouse model of a low level of chronic ethanol consumption used in previous studies (Cuzon et al., 2008; Skorput and Yeh, 2016).

Twenty-four hours after incubation, the slice cultures were washed in PBS and immerse-fixed in 4% paraformaldehyde/0.1 m PBS overnight at 4°C. Following cryoprotection in 30% sucrose/0.1 m PBS, the slice cultures were mounted on charged slides and coverslipped with FluorSave Reagent (Calbiochem).

### Electrophysiology

On E16.5, pregnant dams were euthanized with CO_2_ asphyxiation, and fetuses were removed by caesarian section. UV goggles were used to visualize Nkx2.1Cre/Ai14 embryos, which express tdTomato fluorescence in the cortical regions. The brains expressing tdTomato fluorescence were dissected in ice-cold oxygenated artificial CSF (aCSF) containing the following: 125 mm NaCl, 2.5 mm KCl, 1 mm MgCl_2_, 1.25 mm NaH_2_PO_4_, 2 mm CaCl_2_·2H_2_O, 25 mm NaHCO_3_, and 25 mm D-glucose, pH 7.4 (adjusted with NaOH). The brains were embedded in 3.8% low-melting point agarose (Invitrogen). Coronal telencephalic slices (250 μm) containing the embryonic medial prefrontal cortex (mPFC) were obtained using a vibratome (Electron Microscopy Services) and immersed in cutting solution containing the following: 3 mm KCl, 7 mm MgCl_2_, 1.25 mm NaH_2_PO_4_, 0.5 mm CaCl_2_, 28 mm NaHCO_3_, 5 mm D-glucose, and 110 mm sucrose, pH 7.4 (adjusted with 1 N NaOH). The slices were then stored in a reservoir of aCSF at room temperature. Only slices from the rostral telencephalon with the embryonic mPFC clearly discernable were used for recording.

An acute 250-μm telencephalic slice was transferred to a recording chamber and stabilized by a platinum ring strung with thin plastic threads. The slice was maintained at 34°C on a heated stage fit onto a fixed-stage upright microscope (BX51WI, Olympus) and was perfused at a rate of 0.5–1.0 ml/min with oxygenated aCSF. Nkx2.1^+^ GINs were identified using a 40× water immersion objective (Olympus) under fluorescence illumination and Hoffman Modulation Optics (Modulation Optics). Images were displayed on a computer monitor through a video camera (Integral Technologies), which aided the navigation and placement of the recording and drug pipettes.

Recording pipettes for whole-cell patch clamp recording were pulled from borosilicate glass capillaries (1.5-mm outer diameter, 0.86-mm inner diameter; Sutter Instrument Co). The pipettes were back-filled with KCl internal-solution containing the following: 140 mm KCl, 2 mm CaCl_2_, 2 mm MgCl_2_, 11 mm EGTA, and 10 mm HEPES, pH 7.4. The patch pipettes had resistances of 8–10 MΩ. Recordings were made using an AxoPatch 700B amplifier (Molecular Devices Inc.). Membrane currents were digitized at 20 kHz (Digidata 1320A; Molecular Devices), recorded with low-pass filtering at 10 kHz. Recordings were analyzed offline using Mini Analysis (Synaptosoft) and Clampfit 10.3 (Skorput et al., 2019).

Nifedipine, TEA, and TTX were dissolved in aCSF immediately before recording to a working concentration of 20 μm, 20 mm, and 1 μm, respectively. Ethanol was prepared fresh by diluting 95% ethanol with aCSF to 18 mm for the electrophysiological experiments, which is equivalent to the blood alcohol concentration of 80 mg/dl BAL attained in the binge-ethanol exposure paradigm used in this and previous studies (Skorput et al., 2015, 2019).

Drug solutions were loaded into separate barrels of an eight-barrel drug pipette assembly that was placed within 10 μm of the soma of the cell under study and applied using regulated pulses of pressure (≤3 p.s.i.; Picospritzer, General Valve Corporation). The timing and duration of the pressure pulses were controlled by a multichannel timing unit and pulse generator (Pulsemaster A300, WPI). One of the barrels of the multibarrel drug pipette was filled with aCSF, which was applied between drug applications to clear drugs from the vicinity of the cell and to control for mechanical artifacts that can occur because of bulk flow.

### Processing of embryonic tissues

Time-pregnant dams were euthanized by CO_2_ asphyxiation on E16.5, following control, ethanol (5% w/w), nifedipine (0.15 mg/kg body weight) + ethanol (5% w/w), or nifedipine (0.15 mg/kg body weight) treatment. The embryos were removed, their brains dissected, and immerse-fixed in 4% PFA/0.1 m PBS overnight at 4°C. Following cryoprotection in 30% sucrose/0.1 m PBS overnight, 30-μm cryosections were cut with a sliding microtome. The slices were mounted on charged glass slides, DAPI counterstained, and coverslipped with FluorSave Reagent (Calbiochem).

For immunohistochemistry, 30-μm coronal sections were collected into PBS and washed overnight at 4°C. The sections were then blocked for 30 min at room temperature in PBS containing 10% normal goat serum (NGS) and 0.025% Triton X-100. The sections were then incubated overnight at 4°C with rabbit anti-Cav1.2 primary antibody (Alomone Labs) at a dilution of 1:400 in PBS. The sections were washed in PBS 2× and incubated overnight with a 1:1000 dilution of Alexa Fluor 488-conjugated goat-anti-rabbit secondary antibody (Invitrogen) in PBS. Negative control without primary antibody was routinely processed in parallel.

### Immunofluorescence imaging and analysis

Fluorescent images were acquired using a CCD camera (Hamamatsu) fit onto a spinning disk confocal microscope (BX61WI; Olympus) and CellSens software (Olympus). Images were montaged using Photoshop to yield a full view of the region of interest.

For each embryonic brain, 10 consecutive sections of the embryonic mPFC beginning at equivalent rostral-caudal levels were imaged and analyzed for counts of Nkx2.1^+^ GINs. For each litter, sections from a minimum of three brains were imaged. The embryonic mPFC was delineated as part of the dorsomedial telencephalon based on DAPI counterstaining of the sections used for analyzing cells. Nkx2.1^+^ GINs were manually counted using Fiji’s cell counting tool by trained experimenters blinded to the experimental condition.

For organotypic slice cultures, images were montaged with Photoshop to allow visualization of the cortex throughout its thickness, from the corticostriate juncture (CSJ) to the dorsal apex. One 200-μm bin immediately proximal to the CSJ, was delineated as one of the regions of interest. Distal to the CSJ, five consecutive 100-μm bins spanning the thickness of the cortex were organized (Cuzon et al., 2006). The Nkx2.1^+^ GINs were manually quantified by trained experimenters blinded to the experimental condition using Fiji’s cell counting tool. Cell counts in each 100-μm bin were normalized by counts from the 200-μm bin proximal to the CSJ from each tissue to calculate the crossing index (Cuzon et al., 2006).

### Statistics

For histologic analyses, *n* represents the number of litters to minimize litter effects. All groups of histologic data were acquired from ten 30-μm tissue sections per animal from a minimum of three individual animals from multiple litters. For electrophysiological experiments, *n* also refers to the number of litters used. All statistical analyses were conducted using GraphPad Prism (version 5.0). Power calculations were performed using the G*Power 3.1 software depending on whether the data were analyzed using *t* test or ANOVA. Variance and expected differences were estimated by using group means and standard deviations from preliminary data or past experience in similar studies and reviews of related use in the literature. Group means were compared by paired *t* test, one-way ANOVA or two-way ANOVA with appropriate *post hoc* test as indicated, and reported in Results as mean (10) ± SE.

## Results

### Binge exposure to ethanol does not affect Cav1.2 expression levels in the embryonic PFC

**Figure 1.**
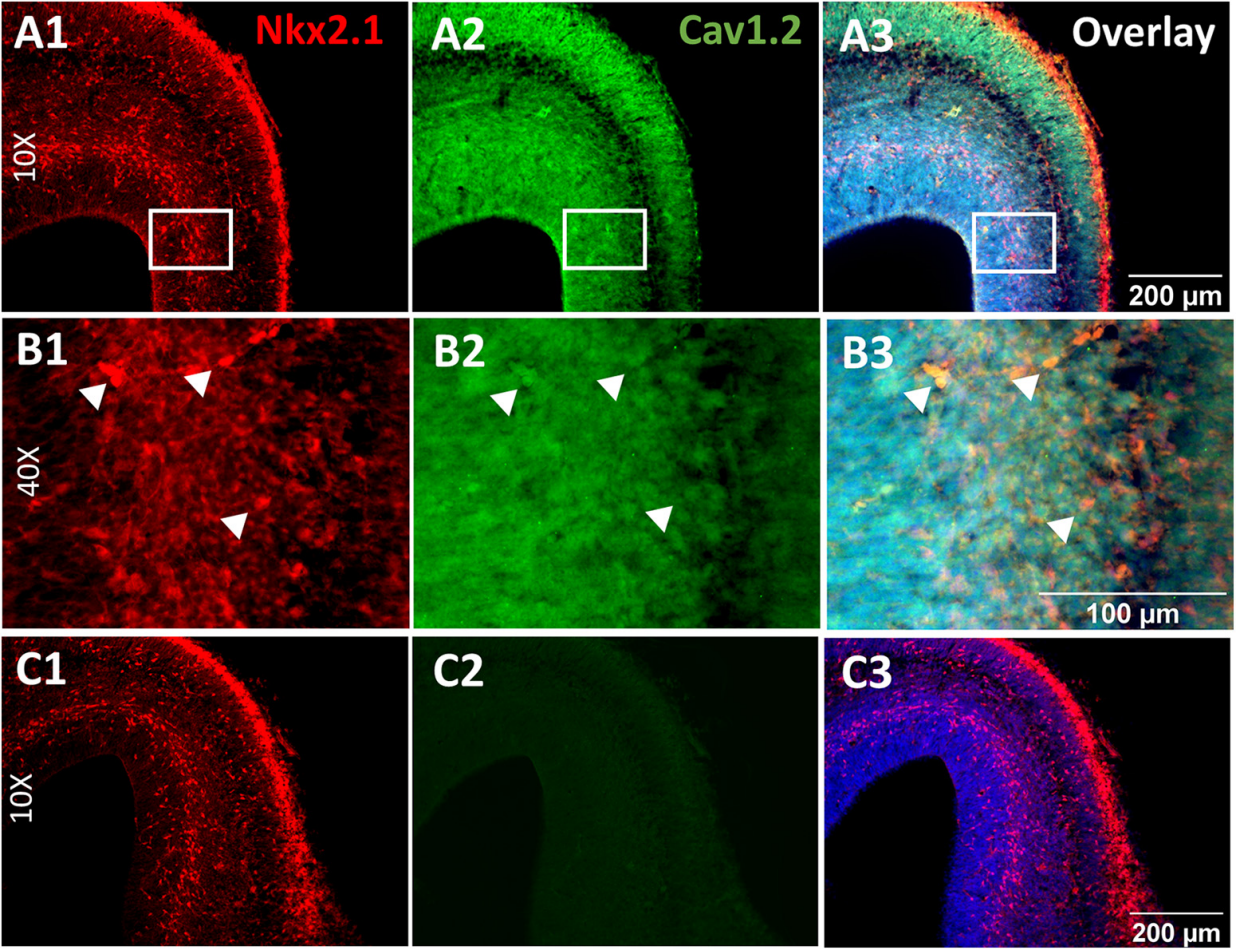
Cav1.2 is expressed in the embryonic mPFC. ***A***, Representative images of histologic sections from Nkx2.1/Ai14 E16.5 mouse brain (***A1***) immunostained for Cav1.2 (***A2***) and overlayed with images of DAPI counterstaining and Nkx2.1/tdTomato-fluorescent GINs (***A3***). Images were captured at 10× magnification on a spinning disk confocal microscope. ***B***, Representative images at 40× magnification. These images are magnified images of the area demarcated by the white box in ***A1–A3***. ***C***, Representative images of no primary antibody negative control of Cav1.2 staining.

**Figure 2.**
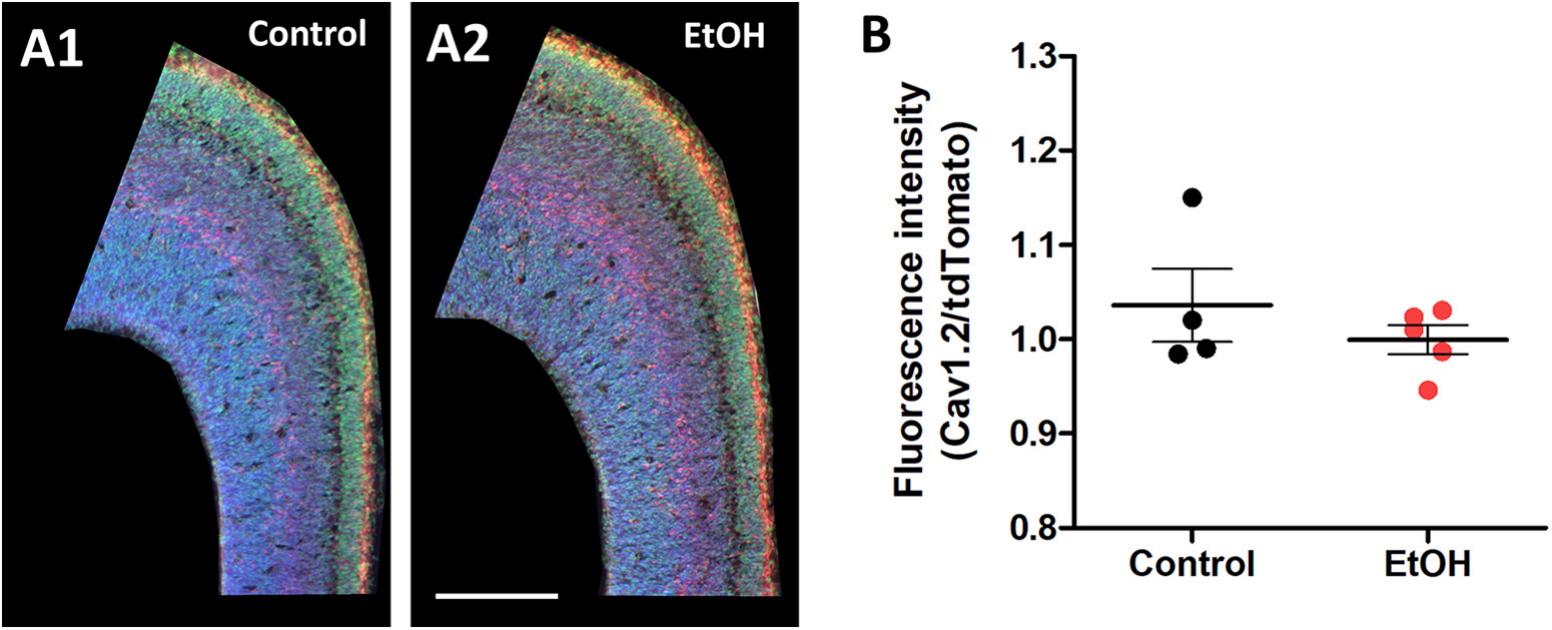
Prenatal ethanol exposure does not alter Cav1.2 expression. ***A***, Representative images of Cav1.2 staining overlayed with DAPI and Nkx2.1 in the mPFC of control (***A1***) and ethanol-fed (***A2***) E16.5 mouse brain. ***B***, Quantification of fluorescence intensity ratio of Cav1.2 to Nkx2.1 in the mPFC of control and ethanol-treated mice. Unpaired *t* test. For statistical details, see Results.

We first asked whether LTCCs are expressed in Nkx2.1^+^ GINs as they migrate tangentially and become positioned in the cortical plate of the embryonic mPFC. Immunohistochemical staining of Cav1.2 in sections from Nkx2.1Cre-Ai14 mouse at E16.5 showed that virtually all cells express Cav1.2, including the Nkx2.1^+^ GINs (Fig. 1). We then assessed whether prenatal ethanol exposure alters the expression levels of Cav1.2 in the embryonic mPFC. To this end, pregnant dams were fed liquid diet containing 5% ethanol or isocaloric control from E13.5-E16.5. Embryonic brains dissected at E16.5 were stained for Cav1.2, and immunofluorescence intensity of Cav1.2 and tdTomato was measured using a spinning disk confocal microscope. Fluorescence intensity of Cav1.2 was normalized to that of tdTomato, which is expressed uniformly in the Nkx2.1Cre-Ai14 mice. There was no difference between the fluorescence intensity ratio between control and ethanol-treated embryos at E16.5, indicating that the binge exposure paradigm does not alter the expression levels of Cav1.2 (control; 1.07 ± 0.08, *n* = 4 litters; ethanol; 1.00 ± 0.027, *n* = 5 litters; unpaired *t* test, *p* > 0.999; Fig. 2).

### Nifedipine co-treatment *in vitro* prevents the ethanol-induced aberrant tangential migration

**Figure 3.**
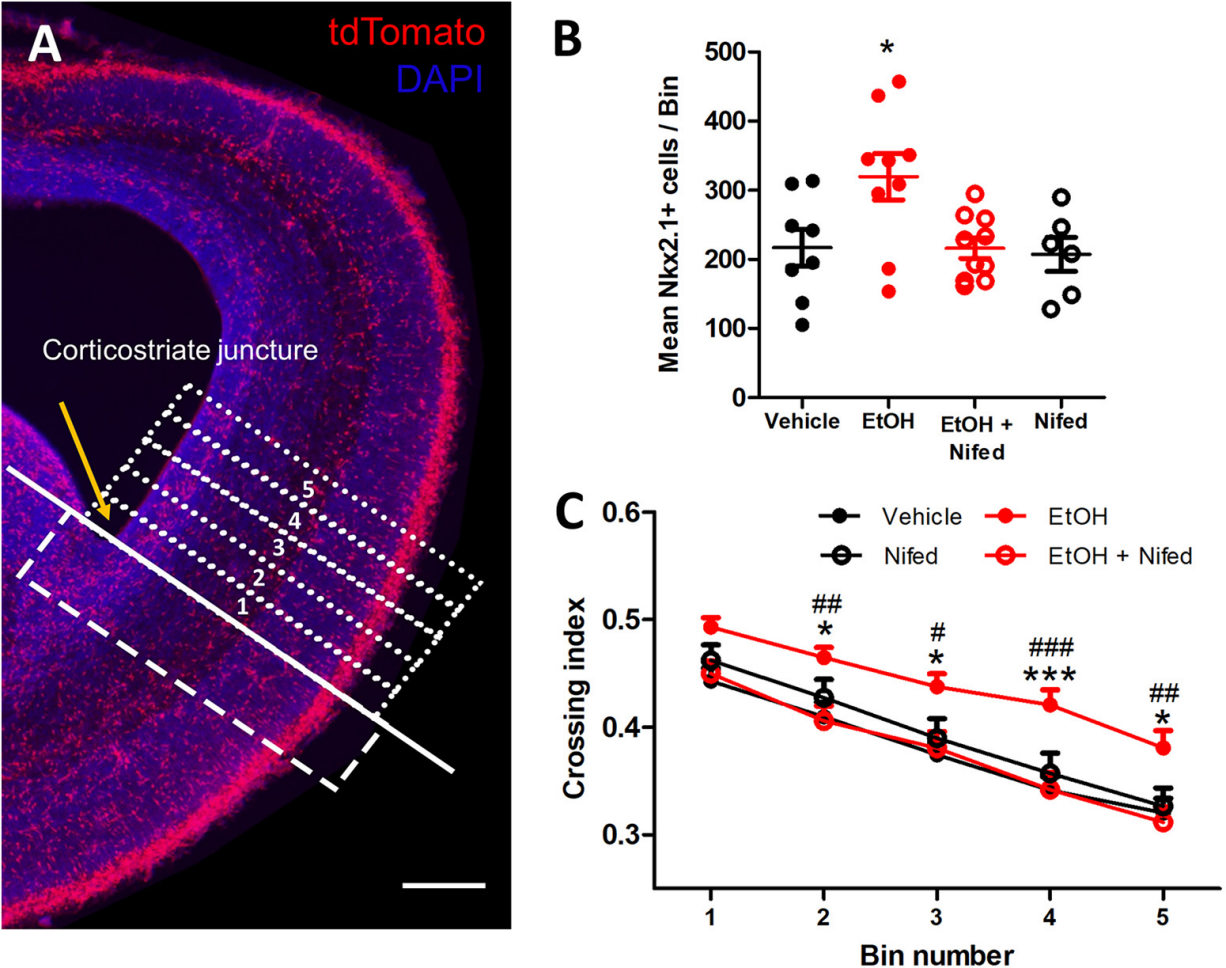
Nifedipine prevents ethanol-induced aberrant migration in organotypic slice cultures. ***A***, Representative image of Nkx2.1/Ai14 embryonic mouse brain with five bins (100 μm wide each) above CSJ and one 200-μm bin below CSJ. ***B***, Quantification of mean Nkx2.1^+^ cells per individual bins in control (vehicle; 3 litters, 3 females, 5 males), ethanol (EtOH; 3 litters, 6 females, 3 males), ethanol + nifedipine (EtOH + Nifed; 3 litters, 7 females, 3 males), and nifedipine (Nifed; 3 litters, 2 females, 4 males) treated organotypic slice cultures. ***C***, Quantification of crossing index for control (vehicle), ethanol (EtOH), ethanol + nifedipine (EtOH + Nifed), and nifedipine (Nifed) treated organotypic slice cultures; * compared with control, # compared with EtOH + Nifed. *,#*p* < 0.05, **,##*p* < 0.01, ***,###*p* < 0.001, two-way ANOVA with Tukey’s *post hoc* test.

To determine the involvement of LTCCs in ethanol-induced aberrant tangential migration, organotypic slice cultures containing the embryonic mPFC were prepared from E14.5 Nkx2.1Cre-Ai14 brains. The slices were incubated in either control or 6.5 mm ethanol-containing medium without or with 20 μm nifedipine. Following 27 h of incubation, the slices were fixed in 4% paraformaldehyde/PBS and tangential migration of MGE-derived interneurons was subsequently assessed by counting the number of cells in consecutive 100-μm bins in the mPFC beginning at the CSJ. The number of cells in each bin was normalized to the number of cells in the 200-μm bin delineated immediately proximal to the CSJ to calculate the crossing index (Fig. 3*A*). When the number of Nkx2.1^+^ GINs per 100-μm bin was compared among the different treatment groups, ethanol-treated organotypic slice cultures had significantly higher number of cells compared with those in the control group (vehicle; 216.9 ± 26.69 cells *n* = 8 cultures, EtOH 319.6 ± 33.54 cells, *n* = 9 cultures, one-way ANOVA, Tukey’s *post hoc* test, *p* < 0.05; Fig. 3*B*). On the other hand, the number of Nkx2.1^+^ GINs in the organotypic cultures co-treated with LTCC blocker nifedipine and ethanol were similar to that of control (Nifed + EtOH; 216.1 ± 14.71 cells, *n* = 10 cultures, *p* > 0.999). At the concentration of nifedipine used (20 μm), treatment with the LTCC blocker alone did not alter the density of Nkx2.1^+^ GINs in the cortex compared with controls (Nifed; 207.2 ± 24.69, 6 cultures; *p* > 0.999). When the crossing index was analyzed with two-way ANOVA, the number of Nkx2.1^+^ GINs was significantly higher in ethanol treated cultures compared with control in cortical regions more distal to the CSJ (Fig. 3*C*). Cultures treated with nifedipine alone and those co-treated with nifedipine and ethanol resulted in a similar crossing index as that of control in all bins. These results implicate calcium flux through the LTCCs being involved in the ethanol-induced aberrant tangential migration, and that blocking LTCCs with nifedipine can normalize the effect of ethanol.

### Nifedipine co-treatment *in vivo* prevents the ethanol-induced aberrant tangential migration

**Figure 4.**
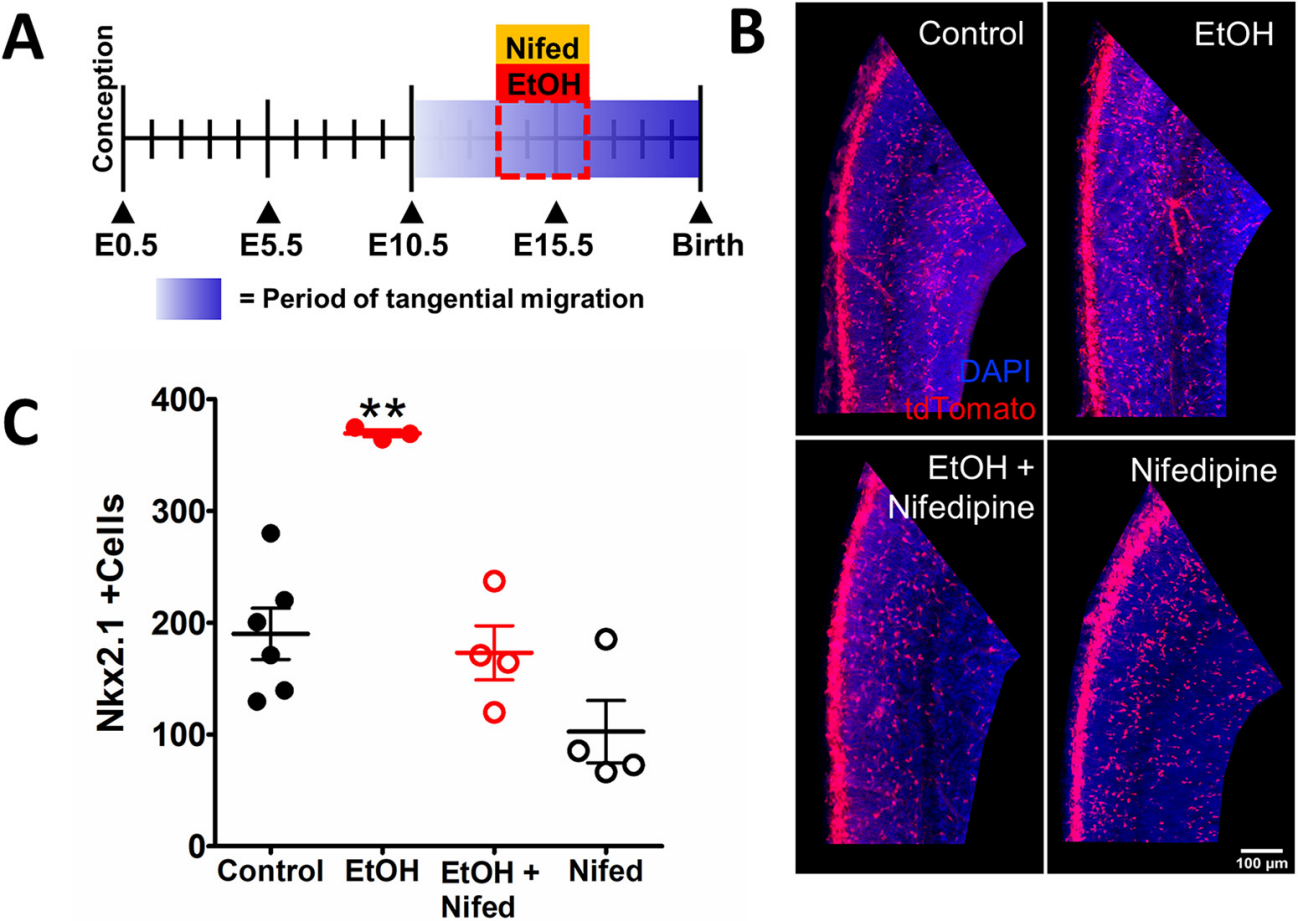
Maternal nifedipine treatment normalizes ethanol-induced enhancement of tangential migration *in vivo*. ***A***, Experimental timeline. Graded blue box highlights the period of tangential migration, beginning at ∼E01.5 and tapering out by ∼P0. Binge-type ethanol exposure and nifedipine co-treatment begins on E13.5 and ends on E16.5 (red dashed lines). ***B***, Fluorescent images of mPFC counterstained with DAPI in mice that received control, binge-type maternal ethanol consumption from E13.3 to E16.5 (EtOH), ethanol exposure in combination with maternal nifedipine treatment in liquid diet (0.15 mg/kg body weight; EtOH+Nifed), or nifedipine only treatment (Nfed). Scale bars: 100 μm. ***C***, Quantification of Nkx2.1^+^ cells in all treatment groups; **p < 0.01 compared with control, one-way ANOVA with Tuckey’s *post hoc* test.

To investigate whether the preventive effect of nifedipine when co-treated with ethanol seen *in vitro* can be replicated *in vivo*, we fed pregnant dams harboring Nkx2.1Cre-Ai14 embryos with a liquid diet containing ethanol (5% EtOH w/w) as well as nifedipine (0.15 mg/kg body weight dissolved in DMSO) from E13.5 to E16.5 according to the timeline outlined in Figure 4*A*. The dose of nifedipine we used was based on the medical dose of 10 mg prescribed to pregnant women for emergency hypertension and that of the average body weight for first trimester pregnant women is 79 kg (ACOG Committee Opinion, 2019). On E16.5, we analyzed the number of Nkx2.1^+^ GINs in the embryonic mPFC of the progeny (Fig. 4*B*,*C*). The laminar localization of these neurons in the embryonic mPFC was not systematically analyzed. Ethanol treatment significantly increased the number of Nkx2.1^+^ GINs in the mPFC (control; 190.9 ± 22.87 cells, *n* = 6 litters, EtOH; 369.3 ± 3.063 cells, one-way ANOVA, Tukey’s *post hoc* test, *p* < 0.0005, *n* = 3 litters; Fig. 4*C*). Nifedipine treatment during the binge-exposure to alcohol attenuated the number of Nkx2.1^+^ GINs to a level similar to that of control (Nifed + EtOH; 173.0 ± 24.20 cells; *p* > 0.99, *n* = 4 litters). Nifedipine alone, at the concentration used in this study, did not alter the tangential migration of GINs into the mPFC compared with controls (Nifed; 102.4 ± 27.88 cells; *p* > 0.99, *n* = 4 litters). Thus, the effect of nifedipine in preventing the ethanol-induced enhancement of Nkx2.1^+^ GINs seen *in vitro* in organotypic slices is recapitulated *in vivo.*

### Ethanol exposure increases L-type calcium channel activity

**Figure 5.**
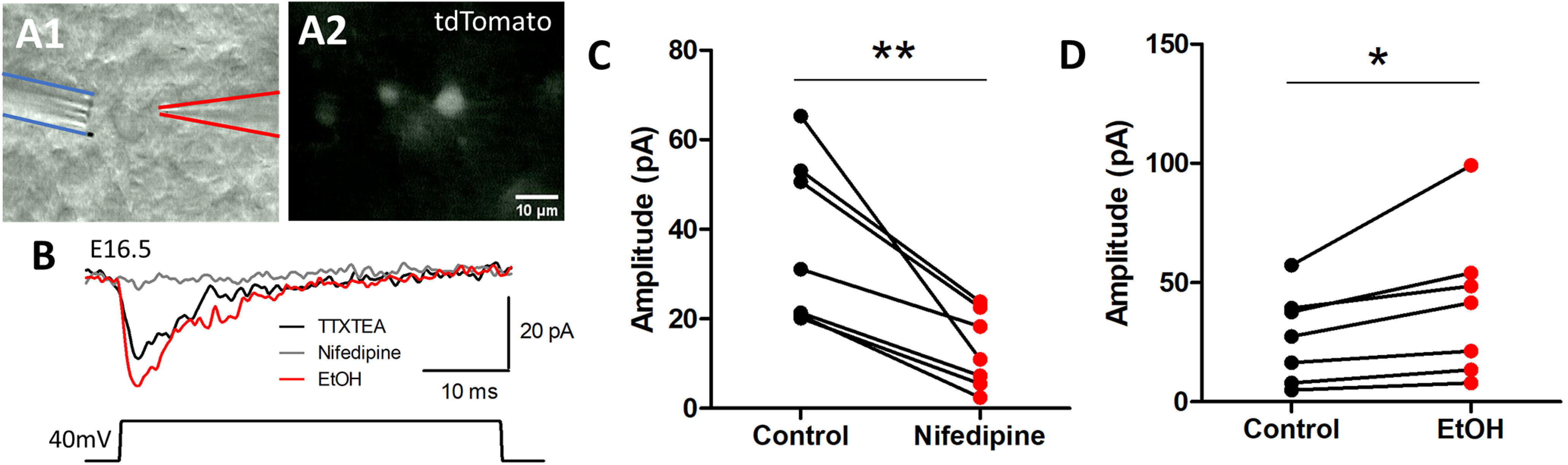
Ethanol acutely potentiates calcium current mediated by L-type calcium channels. ***A***, Representative images of the recording setup. Hoffman modulation contrast image of an acute slice from E16.5 mouse brain with the patch clamp recording pipette (***A1***, right) pointed at a tdTomato-fluorescent Nkx2.1^+^ GIN to be recorded in the whole-cell voltage clamp configuration. A multibarrel drug pipette is navigated to the vicinity of the cell being recorded (***A1***, left). ***B***, Calcium current mediated by LTCCs was isolated by performing 40-mV depolarizing voltage steps in the presence of TTX, TEA (black trace). This current was subtracted with current obtained from 40-mV depolarizing steps in the presence of TTX, TEA, and nifedipine (nifedipine, gray trace). L-type calcium current was measured again in the presence of 18 mm ethanol (EtOH, red trace). ***C***, Quantification of peak amplitude of calcium current mediated by LTCC in the presence of TTX and TEA (control) and nifedipine (nifedipine); **p < 0.01 paired *t* test. ***D***, Peak amplitude of calcium current mediated by LTCC in the before (control) and during acute 18 mm ethanol exposure (EtOH); *p < 0.05 unpaired *t* test.

We next assessed whether ethanol exposure directly affects LTCC-activated currents in tangentially migrating GINs. We performed whole-cell patch clamp recordings from Nkx2.1^+^ GINs in acute slices from the embryonic (E16.5) Nkx2.1/Ai14 mouse brains. A multibarrel drug pipette was used to focally apply 18 mm ethanol, 20 μm nifedipine, 1 μm TTX, 20 mm TEA, and aCSF to cells being recorded from (Fig. 5*A*). Calcium current mediated by LTCCs was isolated by applying a depolarizing +40 mV voltage step from a holding potential of −60 mV. Nifedipine application blocked 65.35% of the inward current (control; 37.49 ± 7.025, nifedipine; 12.99 ± 3.240, *n* = 7 litters, *p* < 0.01; Fig. 5*C*). The inward current mediated through nifedipine-sensitive LTCCs was isolated by subtracting the current recorded in presence of nifedipine from that without nifedipine. This current was measured again before and during acute 18 mm ethanol application (Fig. 5*B*). We found that acute ethanol exposure potentiated the LTCC-activated current amplitude by 33.16% (control; 27.31 ± 7.158, ethanol; 40.86 ± 11.81, *n* = 7 litters, paired *t* test, *p* < 0.05; Fig. 5*D*). These results indicate that ethanol augments LTCC-mediated currents in embryonic Nkx2.1^+^ GINs and, thus, calcium influx consequent to an ethanol-induced potentiation of GABA_A_ receptor-mediated depolarization (Skorput et al., 2019).

## Discussion

We sought to contribute to elucidating the mechanisms underlying the embryonic etiology of FASD in this study. This was motivated by our previous studies that showed ethanol altering tangential migration of GINs (Cuzon et al., 2008; Skorput et al., 2015, 2019). The major findings of this study are three-fold. First, we showed that Cav1.2 (or α1_C_) is ubiquitously expressed in the embryonic mouse cortex. Second, nifedipine, presented either directly to organotypic slices *in vitro* or through *in vivo* treatment of ethanol-consuming pregnant dams, prevents the ethanol-induced aberrant tangential migration in the fetal cortex. Third, we provide evidence that ethanol enhances calcium influx through LTCCs in migrating embryonic GINs. Collectively, these findings provide evidence for calcium and LTCCs being important regulators of the ethanol-induced aberrant tangential migration of GINs in the embryonic mPFC.

**Figure 6.**
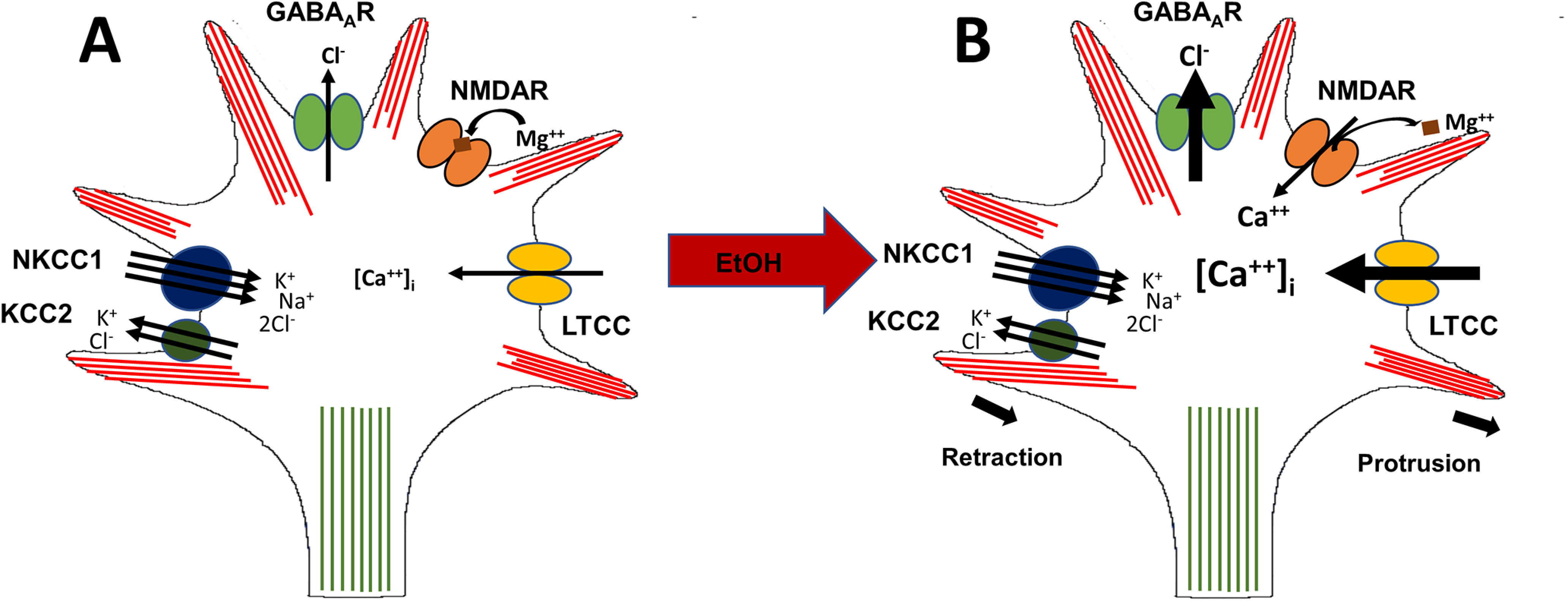
Schematic diagram summarizing the effect of ethanol on migrating embryonic cortical GINs. ***A***, The depolarizing action of GABA in embryonic cortical GINs is established by the predominant expression of NKCC1 chloride importer relative to that of the KCC2 chloride exporter, resulting in heightened intracellular chloride. GABA binding to its cognate GABA_A_ receptor thus causes chloride efflux and membrane depolarization. ***B***, Following ethanol exposure, chloride efflux from GABA_A_ receptors is enhanced, shifting the membrane potential of embryonic GINs to more depolarized levels. This enhanced depolarization of the membrane potential promotes the activation of LTCCs. It is postulated that the increased depolarization may also activate NMDA receptors by releasing the voltage-dependent Mg*^++^* block. The increase in the activity of calcium-permeable channels may, in turn, increase calcium influx, raising intracellular calcium, and further activate downstream calcium signaling mechanisms. The net result of this chloride-to-calcium interplay mobilizes changes in the actin-microtubule dynamics and promote migration of cortical GINs.

Figure 6 integrates the results of the present study and those of a recent one (Skorput et al., 2019) that, together, led to formulating an LTCC-based subcellular mechanistic scheme by which ethanol may mobilize tangential migration in embryonic GINs. Under normal control conditions (Fig. 6*A*), the preponderance of the NKCC1 chloride importer vis-à-vis the KCC2 chloride exporter generates a net high intracellular level of chloride that maintains a depolarized membrane potential in embryonic GINs. In addition, tonic activation of GABA_A_ receptors by an ambient presence of GABA in the extracellular milieu (Cuzon et al., 2006) leads to a net chloride efflux and depolarization of the membrane. Ethanol exposure (Fig. 6*B*) does not directly affect the action of the NKCC1 cotransporter but potentiates chloride efflux through GABA_A_ receptors, resulting in enhanced depolarization (Skorput et al., 2019), which goes on to promote downstream processes that either enhance LTCC activity (Fig. 6*B*) or trigger other mechanisms that increase intracellular calcium dynamics. One such mechanism, as postulated in Figure 6, may be a surrogate activation of the calcium-permeable NMDA receptors following the depolarization-dependent release of the magnesium block. Whether and how such surrogate interacts and comes into play in conferring ethanol’s influence on the tangential migration of cortical GINs await experimental elucidation.

Our results indicate that, although the embryonic neocortex expresses Cav1.2, or α1_C_, at E16.5, prenatal exposure to ethanol did not affect Cav1.2 expression levels in the embryonic PFC. It should be noted that this finding deviates from earlier studies in which chronic ethanol exposure was shown to increase calcium uptake through LTCCs, presumably through the increase in expression of α1 and α2/δ1 subunits of LTCCs in cortical neurons and α1_C_, α2, and β1_b_ in neural crest-derived cell line PC12 (Katsura et al., 2006; Walter et al., 2000). A parsimonious explanation to account for the apparent discrepant results regarding LTCC expression might be because of the use of high doses of ethanol in the previous studies (50–150 mm) vis-à-vis the more moderate concentrations of ethanol (6.5 and 18 mm) used here. In addition, the earlier studies employed a chronic ethanol exposure paradigm (Katsura et al., 2006; Walter et al., 2000), and this differs from the binge-type ethanol exposure early in gestation employed in our study.

Nifedipine treatment in ethanol-exposed organotypic slice cultures prevented the aberrance in tangential migration. Maternal nifedipine treatment along with binge exposure to ethanol also normalized migration disrupted by ethanol *in vivo*. Nifedipine, as a dihydropyridine calcium channel blocker, can have wide-ranging systemic effects, including activation of the reflex sympathetic nervous system, increase in myocardial oxygen supply, and decrease in blood pressure (Gibbons et al., 2003; Abrams et al., 2007). Pregnant mice treated with nifedipine may be subject to such physiological changes, independent of or in addition to its effects on the embryonic central nervous system. However, we favor the prospect that nifedipine’s effect on the tangential migration of Nkx2.1^+^ GINs most likely involves a direct effect, vis-à-vis, secondary effects on physiological alterations, as similar outcomes were recapitulated in our organotypic slice culture experiments that would have arguably circumvented any direct systemic effects.

In the present study, acute exposure to ethanol enhanced the activity of LTCCs. We note that acute ethanol exposure has been shown to inhibit the function of LTCCs in several neuronal preparations (Wang et al., 1991, 1994; Mullikin-Kilpatrick and Treistman, 1995; Zucca and Valenzuela, 2010; Mah et al., 2011; Morton and Valenzuela, 2016). Such differences may arise from the fact that embryonic neurons have relatively depolarized resting membrane potentials (approximately −40 mV; Skorput et al., 2019), such that membrane depolarization exerted by traditionally hyperpolarizing neurotransmitters such as GABA might be more conducive to reaching the activation potential for LTCCs (Nakanishi and Okazawa, 2006; Horigane et al., 2019). We hypothesized that ethanol, by potentiating depolarization through GABA_A_ receptors, may further activate LTCCs, increasing calcium influx into the migrating embryonic GINs (Fig. 6*B*). This is in line with reduced calcium signaling observed in tangentially migrating interneurons with KCC2 upregulation, which decreases GABA-induced depolarization (Bortone and Polleux, 2009). In this study, we did not confirm the role of GABA as a mediator of the observed increase in calcium influx through LTCCs. Nonetheless, our findings provide the basis for investigating further the interplay between GABA_A_ receptor and LTCCs activation in embryonic Nkx2.1^+^ GINs on ethanol exposure.

L-type calcium channels are predictably not the only voltage-gated calcium channels that immature GABAergic cortical interneurons express. Thus, the involvement of L-type calcium channels does not preclude the role of other voltage-activated calcium channels in regulating the tangential migration of Nkx2.1^+^ GINs. Ligand-gated ion channels other than GABA_A_ receptors may also play a role. For example, NMDA receptors are expressed in immature GABAergic cortical interneurons (Soria and Valdeolmillos, 2002). Since NMDA receptor activation relies on membrane depolarization-induced release of magnesium block, a GABA-mediated depolarization may facilitate NMDA receptor activation and, thereby, promote calcium influx in immature GINs. This surrogate activation of NMDA receptors may be augmented with ethanol exposure (Fig. 6). Future investigative work will need to confirm the potential involvement of NMDA and interplay with GABA_A_ receptor activation in this mechanistic signaling scheme.

The present study demonstrated that ethanol increases LTCC-mediated channel activity (Fig. 6*B*). However, how this would enhance tangential migration of cortical GINs still remains unresolved. Calcium signaling is critical in activating the downstream cytoskeletal dynamics in mechanisms underlying neuronal migration (Gomez and Spitzer, 1999; Lautermilch and Spitzer, 2000; Gomez et al., 2001; Wen et al., 2004; Kerstein et al., 2017). Live imaging studies addressing changes in filopodia motility, somal translocation, or trailing tail retraction, as downstream mechanisms of ethanol-induced potentiation of LTCC-mediated calcium current will further elucidate how ethanol affects normal tangential migration of primordial GABAergic cortical interneurons and, thus, contribute to our understanding of the pathoetiology of FASD.

While the diagnosis of FASD occurs postnatally, its embryonic etiology is incompletely understood. The current study focused on investigating how ethanol as a teratogen disrupts the process of migration in the embryonic neocortex. We demonstrated that ethanol increases chloride efflux to enhance GABA-induced depolarization, and increases LTCC activity in migrating interneurons. Ethanol may also activate NMDA receptors, which are calcium permeable, and enhanced activation of voltage-gated calcium channels may increase calcium influx into the cell, which may act on downstream targets to alter cytoskeletal dynamics. Overall, data presented here point to LTCCs playing an important role in the ethanol-induced aberrant tangential migration of cortical GINs. We propose that the inhibition of LTCCs by nifedipine, as an FDA approved drug, may bear therapeutic potential in treating and managing FASD.

## Synthesis

Reviewing Editor: Tod E Kippin, University of California Decisions are customarily a result of the Reviewing Editor and the peer reviewers coming together and discussing their recommendations until a consensus is reached. When revisions are invited, a fact-based synthesis statement explaining their decision and outlining what is needed to prepare a revision will be listed below. The following reviewer(s) agreed to reveal their identity: Mariko Saito, Sandra Mooney.

The present study investigates the contributions of calcium channels to the impact of ethanol on GABAergic neuron disruption during development. The manuscript has been evaluated by two independent reviewers whom both found the study to have significant strengths and relatively few weaknesses. Overall, this study has the potential for significant impact on the field, particularly if the weaknesses raised by the reviewers can be addressed in a revised manuscript.

## Author Response

Dear Dr. Kippin,

We are excited that our manuscript (eN-NWR-0359-21) has been judged to be potentially suitable for publication in eNeuro, pending revisions. We thank you and the reviewers for granting us the opportunity to submit a revision of our manuscript. Along with this cover letter, we submit our revised manuscript entitled “L-Type Calcium Channels Contribute to Ethanol-Induced Aberrant Tangential Migration of Primordial Cortical GABAergic Interneurons in the Embryonic Medial Prefrontal Cortex”.

We read carefully the reviewers’ comments and gave our best attempts to address them in the revised manuscript. Below are point-by-point responses to the reviewers’ comments:

Reviewer 1

Comment: Methods: Although TEA is used in the patch clamp experiment (Figure 5), no information, such as concentrations, is described in Methods. Please add.

Response: The concentration of TEA used in the patch clamp experiment is 20 mM. It is now added in Methods lines 188-189 and Results line 318.

Comment: Figure 1: I suppose that panels A3, B3, and C3 are overlayed images of Nkx2.1, Cav1.2, and DAPI, but the legend of Figure 1 is not clear. Also, the image of C1 seems much lighter than the panel A1. Is it supposed to be similar if the same conditions are applied? Or anti-RFP antibody was used in the panel A1?

Response: We thank the reviewer for the insightful comment. We have now edited the figure legend of Figure 1 (see lines 564-565). In illustrating the images, the fluorescence intensity level was adjusted to be identical in all images. We have now adjusted Figure 1C to be similar to that of A1. We did not use an anti-RFP antibody throughout this study since the tdTomato fluorescence emitted by the GABAergic interneurons in the Nkx2.1/Ai14 cortex was more than sufficiently bright to conduct our analysis without antibody enhancement.

Comment: Figure 4: The authors describe that prenatal ethanol induces “aberrant” GABA neuron migration and show that ethanol induces higher densities of migrating neurons in Figure 3 and 4. Does ethanol only enhance the number of migrating neurons or induce other abnormalities like final localization of these neurons? In Figure 4B, it seems that distributions of migrating GABA neurons are different between control and EtOH, and the distribution pattern of EtOH+Nifedipine group is similar to that of the EtOH group. Is there any EtOH-induced abnormalities that are not restored by nifedipine treatment? The data showing the distribution and densities of Nkx2.1 positive cells in the adult cortices of mice treated prenatally with ETOH or EtOH+Nifedipine may give more information.

It is said that “Nifedipine alone, at the concentration used in this study, did not alter the tangential migration of GABAergic interneurons into the mPFC compared to controls.” (P12 line 305). If LTCC is important in GABAergic interneurons migration, higher concentrations of nifedipine would decrease the migration of the control group?

Response: The data in Figure 3 and 4 took into account the number of cells but we did not analyze final localization in the embryonic layers of the medial prefrontal cortex. A previous study (Skorput et al, 2015) looked at the density distribution and found that the increase in number occurred in marginal zone/cortical plate and intermediate zone, and was trending toward increase in the subventricular/ventricular zone. We have now added a sentence to underscore that the localization of these neurons was not analyzed in the present study (lines 302 - 303).

While we analyzed the differences in the number of cells in the embryonic mPFC, we have not looked at other abnormalities. There indeed may be other ethanol-induced abnormalities that are not restored by nifedipine treatment but we have not confirmed it experimentally. The analysis of whether nifedipine can mitigate the long-term effect of ethanol has yet to be examined. This will be an insightful separate study investigating the adult mPFC following ethanol exposure. The nifedipine concentration used was based on our calculated best estimate of what is administered to humans, and this was stated in our original submission and again in this resubmission (lines 299-302). We did not test higher concentrations in this study.

Comment: Please check references again. I noticed missing of Clancy et al., 2001 and Delatour et al., 2019.

Response: We thank the reviewer for the comment. We have now added the missing references.

Reviewer 2

Comment: The manuscript addresses whether high-voltage activated nifedipine-sensitive L-type calcium channel (LTCC) activity contributes to the pathophysiology of ethanol (EtOH) and finds that the EtOH effects of interest can be blocked with the LTCC blocker, nifedipine. In vivo and in vitro experiments look at migration of GABAergic interneurons and electrophysiology is used to look at function. Unfortunately, there is no experiment showing that activation of LTCCs in the absence of EtOH causes the same outcomes as EtOH. Nor is there any discussion of the published work that shows that EtOH blocks LTCCs.

Response: We thank the reviewer for the thoughtful comment. An experiment to address this comment of activation of LTCCs in the absence of ethanol would be to investigate the effect of BayK on LTCC in neuronal migration. Unfortunately, we did not perform such an experiment. Our proposed model (Figure 6) implies that GABA-induced depolarization may be a mechanism of activating LTCC.

In the original submission, we acknowledged existing literature on ethanol’s effect on LTCC which deviates from what we observed here. We also considered some possibilities that might account for this discrepancy such as differences in the concentration of alcohol and the age of the embryos or neonatal animals. Other possibilities include discrepancies in the route and the timing of administration of alcohol. Additionally, embryonic LTCC properties may actually be different from that expressed in the neonatal or adult brain.

In the Discussion section of this resubmitted manuscript (lines 385-388), we underscore this point and added additional references that are relevant to alcohol’s effect on blocking LTCCs.

Comment: It’s not clear that the word etiology is correctly used - typically, this means cause of or reason for, not underlying mechanism(s). The etiology of FASD is known, this manuscript is examining one of the possible mechanisms.

Response: We have edited the sentence to align with the comments from reviewer. Please see lines 28-29.

Comment: Introduction - first sentence, the word disabilities should be replaced - the facial and growth characteristics aren’t disabilities.

Response: We thank the reviewer for pointing out our oversight. We have changed the word “disabilities” to “abnormalities” in the Abstract as well as Introduction (lines 4 and 40, respectively).

Comment: “Embryonic days 13.5 - 16.5 were operationally defined to be within the age range roughly equivalent to mid-first trimester in humans (Clancy et al., 2001).” - please describe the features of the mouse brain during this time that supports this operational definition.

Response: Indeed, different regions develop at different rates and times during gestation. During embryonic days 13.5 - 16.5, tangential migration of interneuron occurs and the long bone ossification centers appear and the secondary palate closes, similar to what is seen during the mid- and the late-first trimester of humans. We added a reference to strengthen and underscore our point (lines 111-112; lines 449-450, and 528-530).

Comment: A minimum of 3 brains per litter were used for the cell counting experiment - what was the sex distribution? And what was the maximum number of brains from a litter?

Response: 5 - We apologize for the oversight. As we did not genotype for the cell counting experiment, we do not know the sex distribution. A maximum of 4 brains was used per litter.

Comment: For Figure 3, how many animals and litters are represented in each group? What was the sex distribution?

Response: For the vehicle (control) group, there were 2-3 animals from 3 litters. For the ethanol group, there were 3 animals from 3 litters. The nifedipine + ethanol group had 3-4 animals from 3 litters, and the nifedipine group had 2 animals from 3 litters. The sex distribution for this experiment was 18 females and 15 males. (Vehicle, 3 females, 5 males; Ethanol, 6 females, 3 males; Nifedipine + ethanol, 7 females, 3 males; Nifedipine, 2 females, 4 males). This data set is now included in the figure legend of Figure 3 (see lines 580 - 582).

Comment: Why was data from ethanol - nifedipine experiments not tested by 2 factor ANOVA?

Response: The ethanol nifedipine experiment included both cell count experiments and the electrophysiology experiments. We think that the reviewer is referring to the cell count experiment. If so, the in vitro was indeed analyzed by two-way ANOVA (see line 285). The in vivo experiment was analyzed by one-way ANOVA rather than two-way ANOVA because this experiment has only one categorical independent variable (treatment group).

Comment: Increased amplitude in cells during EtOH treatment shown in 5D is subtle for 6 of the 7 cells - would this difference be significant without the one cell that shows an almost doubling of amplitude?

Response: In line with the reviewer’s recommendation, we omitted the data point in question and re-analyzed the data using a two-tailed t-test. We found that the result is still statistically significantly different. We decided to not omit the data point addressed because it is not clear if this cell is an outlier that should be excluded from analysis.

Comment: Similarly, there is 1 cell in 5C that shows a much larger decrease in amplitude - what are the thoughts about these 2 cells that show very different responses?

Response: In Figure 5C, this particular cell also shows a decrease following nifedipine block, but to a greater degree. One potential reason may be that LTCC expression in this cell may be lower and, thus, lead to a greater blocking of LTCC induced by nifedipine.

Comment: Some papers are not in the reference list.

Response: We updated and added citations that appear in the Results and Discussion section. They also now appear in the Reference list. We apologize for the oversight.
